# Manual Thrombus Aspiration and its Procedural Stroke Risk in Myocardial Infarction

**DOI:** 10.1161/JAHA.121.022258

**Published:** 2021-11-15

**Authors:** Yohei Sotomi, Yasunori Ueda, Shungo Hikoso, Daisaku Nakatani, Shinichiro Suna, Tomoharu Dohi, Hiroya Mizuno, Katsuki Okada, Hirota Kida, Bolrathanak Oeun, Akihiro Sunaga, Taiki Sato, Tetsuhisa Kitamura, Yasuhiko Sakata, Hiroshi Sato, Masatsugu Hori, Issei Komuro, Yasushi Sakata, the Osaka Acute Coronary Insufficiency Study (OACIS) Group

**Affiliations:** ^1^ Department of Cardiovascular Medicine Osaka University Graduate School of Medicine Osaka Japan; ^2^ Cardiovascular Division National Hospital Organization Osaka National Hospital Osaka Japan; ^3^ Department of Genome Informatics Osaka University Graduate School of Medicine Osaka Japan; ^4^ Division of Environmental Medicine and Population Sciences Department of Social and Environmental Medicine Graduate School of Medicine Osaka University Osaka Japan; ^5^ Department of Clinical Medicine and Development and Department of Cardiovascular Medicine National Cerebral and Cardiovascular Center Suita Japan; ^6^ School of Human Welfare Studies Health Care Center and Clinic Kwansei Gakuin University Hyogo Japan; ^7^ Osaka International Cancer Institute Osaka Japan; ^8^ Department of Cardiovascular Medicine University of Tokyo Graduate School of Medicine Tokyo Japan

**Keywords:** acute myocardial infarction, percutaneous coronary intervention, stroke, thrombus aspiration, Percutaneous Coronary Intervention

## Abstract

**Background:**

The previous large‐scale randomized controlled trial showed that routine thrombus aspiration (TA) during percutaneous coronary intervention (PCI) was associated with an increased risk of stroke. However, real‐world clinical evidence is still limited.

**Methods and Results:**

We investigated the association between manual TA and stroke risk during primary PCI in the OACIS (Osaka Acute Coronary Insufficiency Study) database (N=12 093). The OACIS is a prospective, multicenter registry of myocardial infarction. The primary end point of the present study is stroke at 7 days. A total of 9147 patients who underwent primary PCI within 24 hours of hospitalization were finally analyzed (TA group, n=4448, versus non‐TA group, n=4699 patients). TA was independently associated with risk of stroke at 7 days (odds ratio [OR], 1.92 [95% CI, 1.19‒3.12]; *P*=0.008) in the simple logistic regression model, while the multilevel random effects logistic regression model with hospital treated as a random effect showed that manual TA was not associated with incremental risk of stroke at 7 days (OR, 0.91 [95% CI, 0.71‒1.16]; *P*=0.435). The 7‐day stroke risk of manual TA was significantly heterogeneous in different institutions (*P*_for interaction_=0.007).

**Conclusions:**

Manual TA during primary PCI for patients with acute myocardial infarction was independently associated with the overall increased risk of periprocedural stroke. However, this result was substantially skewed because of institution specific risk variation, suggesting that the periprocedural stroke may be preventable by prudent PCI procedure or appropriate periprocedural management.

**Registration:**

URL: https://upload.umin.ac.jp/cgi‐open‐bin/ctr_e/ctr_view.cgi?recptno=R000005464. Unique identifier: UMIN000004575.

**Nonstandard Abbreviations and Acronyms**
TA: thrombus aspiration

**Clinical Perspective**

**What Is New?**

- The present study from the large‐scale real‐world East Asian acute myocardial infarction registry showed that manual thrombus aspiration during primary percutaneous coronary intervention for patients with acute myocardial infarction was independently associated with the overall increased risk of periprocedural stroke.
- However, this result was substantially skewed because of institution‐specific risk variation.
- The multilevel random effects logistic regression model with hospital treated as a random effect showed that manual thrombus aspiration was not associated with incremental risk of periprocedural stroke.

**What Are the Clinical Implications?**

- Clinical practice standards at the institutions participating in the registry, as well as the expertise of the interventional cardiologists who performed the procedures, may presumably differ by institutions.
- The skewness observed in the present study may have a strong message that the periprocedural stroke can be preventable by prudent procedure or appropriate periprocedural management.
- Further operator‐oriented and patient‐selective clinical trials would be warranted to accurately investigate the clinical efficacy of manual thrombus aspiration.

Recommendations for routine thrombus aspiration (TA) during primary percutaneous coronary intervention (PCI) in myocardial infarction have been graded as class III in the most recent clinical guidelines.^1^, ^2^, ^3^ This is primarily based on the results from the 2 major randomized trials, TASTE (Thrombus Aspiration in ST‐Elevation Myocardial Infarction in Scandinavia)^4^, ^5^ and TOTAL (Trial of Routine Aspiration Thrombectomy With PCI Versus PCI Alone in Patients With STEMI).^6^, ^7^ These large‐scale randomized trials failed to show the clinical benefits of routine TA during primary PCI in myocardial infarction. Moreover, the TOTAL study showed that routine TA during PCI was even associated with an increased risk of stroke.^6^, ^7^ However, despite such recommendations from the guidelines, not a small number of interventional cardiologists think that manual TA still remains a practically useful tool, presumably because they can feel some benefits from their clinical experiences or generally positive evidences of TA from the previous relatively small studies.^8^, ^9^, ^10^, ^11^

The primary mechanism of stroke was thought to be embolization of thrombus or air to the brain during the procedure.^7^ Therefore, potential mechanisms of increased incidence of stroke associated with manual TA are thought to be closely related to insufficient procedural handling.^7^, ^12^ Given these potential mechanisms, stroke related to manual TA should occur during periprocedural period if it occurs. Furthermore, it must be preventable by prudent PCI procedure.

Several study‐level and patient‐level meta‐analyses concluded that manual TA was associated with an increased incidence of stroke.^6^, ^13^, ^14^, ^15^ However, the findings were always strongly driven by the results of TOTAL study that accounts for more than half of the study population of the meta‐analyses. Moreover, these meta‐analyses mainly focused on the randomized controlled trials rather than real‐world data. Real‐world clinical data with a comparative sample size to the TOTAL study (N=10 064) would be helpful for investigating whether the manual TA is truly related to the increased risk of stroke. In the present study, we aimed to investigate the association between manual TA and stroke risk using a large‐scale Japanese prospective multicenter registry of acute myocardial infarction (AMI) (N=12 093).

## Methods

Our study data will not be made available to other researchers for purposes of reproducing the results because of institutional review board restrictions.

### Study Population

We used the OACIS (Osaka Acute Coronary Insufficiency Study) database (N=12 093) to investigate the impact of manual TA on the risk of stroke in patients with AMI. The OACIS is a prospective, multicenter observational study designed to collect and analyze demographic, procedural, and outcome data in patients with AMI at 25 collaborating hospitals with cardiac emergency units (UMIN000004575). Written informed consent was obtained from each patient. AMI was diagnosed based on the World Health Organization criteria, which requires that at least 2 of the following 3 criteria be met: (1) clinical history of central chest pressure, pain or tightness lasting >30 minutes; (2) ST segment elevation >0.1 mV in at least 1 standard lead; and (3) a rise in serum creatinine phosphokinase concentration to more than twice the normal laboratory value. All collaborating hospitals were encouraged to enroll consecutive patients with AMI. We prospectively collected data obtained by research cardiologists and trained research nurses using a specific reporting form. The OACIS enrolled patients from 1998 to 2014 and followed them up until 2019. The study is registered with the UMIN‐CTR (University Hospital Medical Information Network Clinical Trials Registry) in Japan (ID: UMIN000004575). The study protocol complied with the Helsinki Declaration. The study was approved by the institutional ethics committee of each participating institution. Details of the study design and data collection are described elsewhere.^11^, ^16^, ^17^ In this post‐hoc subanalysis of the OACIS registry, we analyzed patients who underwent primary PCI within 24 hours of hospitalization. Patients who underwent PCI >24 hours after hospitalization, who did not undergo primary PCI, who underwent thrombolysis, or who used percutaneous cardiopulmonary support were excluded from the analysis.

### PCI Procedure and Post‐PCI Medication

Manual TA and other treatment strategies including predilatation, atherectomy, stenting, distal protection, postdilatation, and use of intracoronary imaging were performed at the operators’ discretion. Mechanical TA catheter was not used in the present study. Post‐PCI medications were prescribed at the attending physicians’ discretion. Physicians were encouraged to follow the standard guideline for the treatment of AMI.^3^

### Study End Points

The primary end point of the present study was stroke at 7 days. Secondary end points were stroke from day 7 to hospital discharge, in‐hospital stroke, and stroke after hospital discharge up to final follow‐up.

Stroke was defined as a neurological deficit persisting ≥24 hours attributed to an acute focal injury of the central nervous system by a vascular cause, including cerebral infarction, intracerebral hemorrhage, and subarachnoid hemorrhage. It was diagnosed and adjudicated by on‐site investigators. Information on the clinical event was collected by local investigators when visiting outpatient clinics or through verbal or written contact with patients or family members.

### Statistical Analysis

All analyses were performed using SPSS 26.0 (IBM Corporation, Armonk, NY) or R software (version 4.0.0; R Foundation for Statistical Computing, Vienna, Austria). *P* value of <0.05 was considered statistically significant.

Eligible patients were divided into 2 groups: TA group versus non‐TA group. Data are presented with listwise deletion. Categorical variables are expressed as counts (percentages) and compared with the Chi‐squared test or Fisher exact test. Continuous variables are expressed as mean (SD) or median (interquartile range) and compared using Student *t‐*test or the Mann–Whitney *U*‐test as appropriate. Because excluding missing data cases can cause bias in this analysis and loss of power for detecting a statistical difference, we used the Multivariate Imputation by Chained Equations (MICE) method. The MICE approach is an established imputation method for creating multiple complete data sets in which missing values are replaced with estimates from a specified regression model using the observed data. We created 10 data sets with 20 iterations using the MICE package in R and used 1 randomly selected data set for the following analysis.

As to the in‐hospital stroke events, crude incidence during overall hospitalization was calculated. Hospitalization period was further divided into first 7 days and from 7‐day to hospital discharge. Odds ratios (ORs) with 95% CI were estimated using a simple logistic regression model for these short‐term end points. TA was the variable of interest and the other covariates in the models were as follows: age, sex (men, women), body mass index, comorbidities (hypertension, diabetes, dyslipidemia, chronic kidney disease), history of myocardial infarction, history of cerebrovascular disease, history of cancer, atrial fibrillation, ST elevation and abnormal Q wave on ECG, Killip classification III or IV, pre‐PCI thrombolysis in myocardial infarction grade, target vessel (right coronary artery versus non‐ right coronary artery), distal protection (yes, no), stenting (yes, no), use of intra‐aortic balloon pump, and echocardiographic findings (left ventricular ejection fraction and left ventricular aneurysm). These clinically relevant covariates were selected based on the clinical consensus. Because potential mechanisms of stroke associated with manual TA are thought to be closely related to insufficient procedural handling,^7^, ^12^ it is plausible that risk of stroke is heterogeneous in different hospitals. Therefore, we used multilevel logistic regression models, with hospital treated as a random effect and the abovementioned variables as fixed effect, to consider a possible heterogeneity of stroke risk by manual TA in different institutions. As a sensitivity analysis, different models with a smaller number of essential variables were created to confirm the consistency of the impact of TA on short‐term end points with following variables: age, sex, comorbidities (hypertension and diabetes), history of cerebrovascular disease, history of cancer, atrial fibrillation, Killip classification III or IV, and use of intra‐aortic balloon pump. Subgroup analysis was done according to characteristics of participating institutions (high volume center [>1000 cases versus ≤1000 cases], TA performance rate [>50% versus ≤50%], higher and lower stroke risk [divided by median value of OR for the primary end point]) using the simple logistic regression models. A Forest plot is used to illustrate the treatment effect for the primary end point in different subgroups with interaction. Bonferroni correction was used for the adjustment of multiple comparisons. *P* value of <0.017 was considered statistically significant.

As for the stroke events after hospital discharge up to final follow‐up, differences in survival curves between TA groups versus non‐TA group were estimated by the Kaplan‐Meier method and analyzed using the log‐rank test. Study follow‐up started at the time of hospital discharge. Hazard ratios (HRs) with 95% CI were estimated using the Cox proportional hazards model. TA was the variable of interest and the other covariates in the models were as follows: age, sex (men, women), body mass index, comorbidities (hypertension, diabetes, dyslipidemia, chronic kidney disease), history of myocardial infarction, history of cerebrovascular disease, history of cancer, atrial fibrillation, ST elevation and abnormal Q wave on ECG, Killip classification III or IV, pre PCI thrombolysis in myocardial infarction grade, target vessel (right coronary artery versus non‐right coronary artery), distal protection (yes, no), stenting (yes, no), intra‐aortic balloon pump use, echocardiographic findings (left ventricular ejection fraction, left ventricular thrombus, and left ventricular aneurysm) and medications at hospital discharge (angiotensin‐converting enzyme inhibitor or angiotensin II receptor blocker, and beta blocker]. Anticoagulants and statins were not included in the model because of strong correlations with atrial fibrillation and dyslipidemia, respectively. Since antiplatelet agents were used in most of the cohort, we did not involve the factor in the model. These clinically relevant covariates were selected based on the clinical consensus. The proportional hazards assumption of TA for stroke was confirmed by Schoenfeld residuals (*P*=0.700). A sensitivity analysis was performed with a Fine–Gray model to account for the competing risk of all‐cause death from stroke.^18^ We calculated E‐value to assess how robust associations are to potential unmeasured or uncontrolled confounding.^19^ The E‐value is defined as the minimum strength of association on the risk ratio scale that an unmeasured confounder would need to have with both the treatment and the outcome to fully explain away a specific treatment‐outcome association, conditional on the measured covariates.^19^

## Results

### Patient Demographics

**Figure 1:**
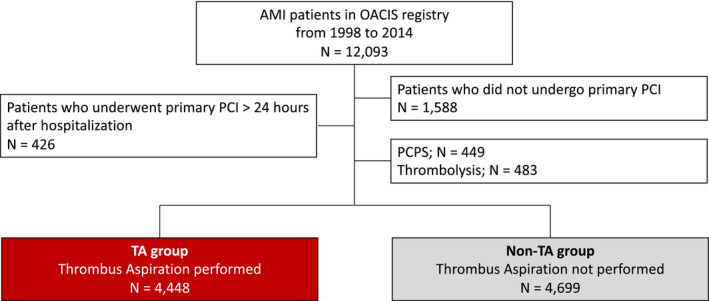
Patient flowchart. AMI indicates acute myocardial infarction; OACIS, Osaka Acute Coronary Insufficiency Study; PCI, percutaneous coronary intervention; PCPS, percutaneous cardiopulmonary support; and TA, thrombus aspiration.

Among the 12 093 patients enrolled between 1998 and 2014, 9147 eligible patients were finally analyzed (Figure 1). The TA group and the non‐TA group consist of 4448 and 4699 patients, respectively. Baseline demographics of patients in the TA and non‐TA groups in the original data set are tabulated in Table 1. Pre‐PCI thrombolysis in myocardial infarction flow grade was worse, stent implantation was more frequently performed, and prescription rates of beta blocker and statin were higher in the TA group than in the non‐TA group. TA performance rate gradually increased in the study period (*P* for trend <0.001; Cochran‐Armitage trend test) (Figure S1). Baseline demographics of patients analyzed and those excluded from the analysis are presented in Table S1.

**Table 1:** Patient Characteristics

	TA group	non‐TA group	*P* value	Missing (%)
Patients, n	4448	4699		
Age, y	66.0 (58.0‒75.0)	67.0 (58.0‒75.0)	0.005	0
Male sex	3458 (77.8)	3522 (75.0)	0.002	0
Diabetes	1361 (31.4)	1662 (36.4)	<0.001	2.7
Hypertension	2719 (62.9)	2767 (60.8)	0.040	3
Dyslipidemia	1938 (45.3)	1976 (44.1)	0.242	4.2
Smoking	2842 (65.2)	2887 (63.2)	0.052	2.4
Chronic kidney disease	286 (6.6)	342 (7.6)	0.056	3.4
Atrial fibrillation	299 (6.8)	236 (5.2)	0.001	2
Prior myocardial infarction	425 (9.7)	610 (13.4)	<0.001	2.3
History of cerebrovascular disease	376 (8.6)	432 (9.6)	0.108	3.4
History of cancer	281 (6.4)	236 (5.3)	0.020	3.4
Systolic blood pressure on admission, mm Hg	136.0 (116.0‒155.0)	138.0 (117.0‒158.0)	0.019	29.3
Diastolic blood pressure on admission, mm Hg	80.0 (68.0‒92.0)	80.0 (68.0‒92.0)	0.349	30.8
Heart rate on admission, bpm	77.0 (64.0≤90.0)	79.0 (65.50‒94.0)	<0.001	29.5
Low‐density lipoprotein cholesterol, mg/dL	121.0 (98.0‒147.0)	119.0 (94.0‒145.0)	0.087	60.3
HbA1c, %	5.60 (5.20‒6.40)	5.60 (5.10‒6.70)	0.995	24.2
Killip class III or IV	315 (7.1)	455 (9.7)	<0.001	0
ST elevation on ECG	3944 (89.6)	3921 (84.9)	<0.001	1.4
Abnormal Q wave on ECG	1907 (44.7)	2153 (47.2)	0.020	3.5
Time from symptom onset to primary PCI, hour	3.50 (2.0‒8.60)	5.0 (2.50‒14.0)	<0.001	13.6
Culprit vessel				
Right coronary artery	1772 (40.4)	1440 (32.5)	<0.001	3.6
Left main trunk or left anterior descending artery	2061 (46.9)	2317 (52.3)	<0.001	3.6
Left circumflex artery	630 (14.4)	727 (16.4)	0.008	3.6
TIMI grade pre PCI			<0.001	15.8
TIMI 0	1979 (58.6)	2260 (52.2)		
TIMI 1	422 (12.5)	443 (10.2)		
TIMI 2	631 (18.7)	857 (19.8)		
TIMI 3	343 (10.2)	771 (17.8)		
Collateral blood flow (+)	1456 (33.6)	1520 (33.0)	0.537	2.2
Distal protection performed	563 (12.7)	138 (2.9)	<0.001	0
Stenting performed	3847 (86.5)	2499 (53.2)	<0.001	0
Post‐PCI laboratory data				
Peak CK, IU/L	2275.0 (1115.0‒4083.0)	1769.50 (828.0‒3437.25)	<0.001	4.2
Peak CK‐MB, IU/L	211.0 (104.0‒380.0)	152.0 (68.0‒287.50)	<0.001	10.8
Medication at discharge				
ACEi or ARB	3489 (81.3)	3219 (73.7)	<0.001	5.3
Beta blocker	2834 (66.1)	2091 (47.9)	<0.001	5.3
Statin	2623 (61.1)	1866 (42.7)	<0.001	5.3
Antiplatelets	4218 (98.3)	4292 (98.3)	0.890	5.3
Anticoagulants	597 (13.9)	772 (17.7)	<0.001	5.3
LVEF	53.55 [45.12, 60.35]	54.61 [45.32, 61.65]	0.008	27.9
LV thrombus	36 (1.1)	43 (1.3)	0.540	28.6
LV aneurysm	47 (1.5)	84 (2.6)	0.003	29.5
Length of hospitalization, d	18.0 [14.0, 25.0]	23.0 [15.0, 31.0]	<0.001	0
Enrollment period, y			<0.001	0
1998–2003	758 (17.0)	2859 (60.8)		
2004–2009	2175 (48.9)	1302 (27.7)		
2010–2014	1515 (34.1)	538 (11.4)		

Data are expressed as median [interquartile range] or number (percentage). ACEi indicates angiotensin‐converting enzyme inhibitor; ARB, angiotensin II receptor blocker; CAG, coronary angiography; CK, creatine kinase; CK‐MB, creatine kinase myocardial band; PCI, percutaneous coronary intervention; TA, thrombus aspiration; and TIMI, thrombolysis in myocardial infarction.

### Stroke

**Figure 2:**
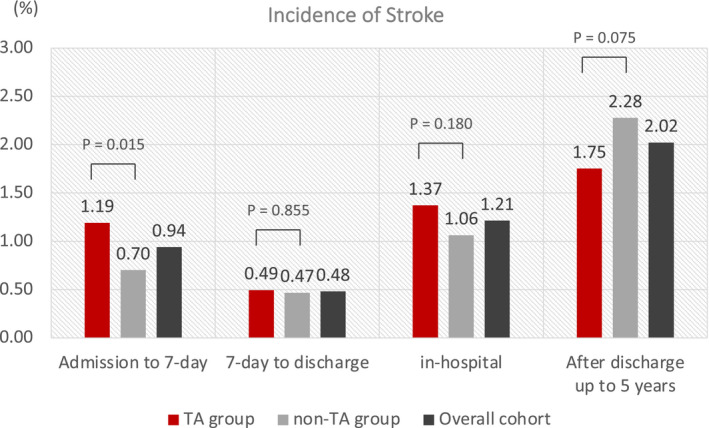
Incidence of the primary and secondary end points. TA indicates thrombus aspiration.

The median length of hospital stay was 18 [14, 25] versus 23 [15, 31] days in the TA group versus non‐TA group, respectively (*P*<0.001). A total of 86 patients experienced the primary end point of stroke during the first 7 days. In this periprocedural period, incidence of stroke was significantly higher in the TA group than in the non‐TA group [53/4448 (1.19%) versus 33/4699 (0.70%), *P*=0.015] (Figure 2). Annual incidence of the primary end point increased as the TA performance rate got higher (*P* for trend=0.031) (Figure S1). There were no significant differences in the secondary end points of stroke from day 7 to hospital discharge, in‐hospital stroke, and stroke after discharge up to final 5‐year follow‐up.

**Figure 3:**
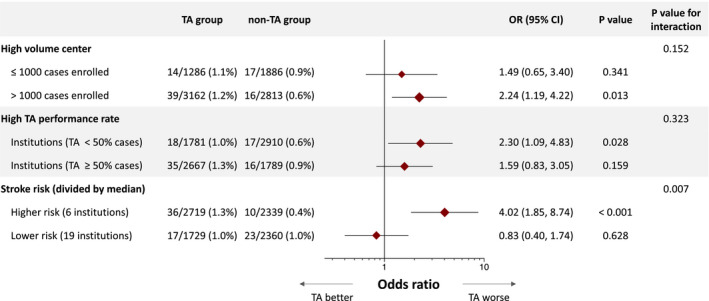
Subgroup analysis. A Forest plot is used to illustrate the primary end point stroke (at 7 days) in different subgroups with interaction for treatment effect assessed. Bonferroni correction was used for the adjustment of multiple comparisons. *P* value of <0.017 was considered statistically significant. OR indicates odds ratio; and TA, thrombus aspiration.

TA was independently associated with risk of stroke at 7 days (OR, 1.92 [95% CI, 1.19‒3.12]; *P*=0.008) in the simple logistic regression model, whereas it was not in the multilevel model (OR, 0.91 [95% CI, 0.71‒1.16]; *P*=0.435) (Table 2). The E‐value to assess the potential effect of unmeasured confounding on the risk assessment for the primary end point was 3.26. TA was not associated with stroke from day 7 to hospital discharge or in‐hospital stroke in both models (Table S2 and S3). These findings were consistent with those presented by different models as a sensitivity analysis (Table S4, S5, and S6). Figure S2 and S3 show scatter plots of 7‐day stroke incidences by institutions. No significant relationship was found between TA performance rate, case numbers enrolled, and incidence of 7‐day stroke. The treatment effect for the primary outcome in several hospital subgroups is shown in Figure 3. It was consistent irrespective of procedural volume and TA performance rate of institutions. However, some specific institutions had substantially high risk of 7‐day stroke (3 institutions showed OR >10). In the low‐risk institutions (divided by the median OR), the incremental risk was not statistically significant (OR, 0.83 [95% CI, 0.40‒1.74]; *P*=0.628), whereas in the high‐risk institutions, manual TA was independently associated with periprocedural stroke (OR, 4.02 [95% CI, 1.85‒8.74]; *P*<0.001) (*P*_for interaction_=0.007). Patient characteristics in the low‐risk and high‐risk institutions are summarized in Table S7.

**Table 2:** Impact of Thrombus Aspiration on the Primary End Point

	Simple logistic regression model	*P* value	Multilevel logistic regression model	*P* value
	Stroke at 7 d		Stroke at 7 d	
	OR (95% CI)		OR (95% CI)	
Thrombus aspiration	1.92 (1.19‒3.12)	0.008	0.91 (0.71‒1.16)	0.435
Male sex	1.12 (0.68‒1.86)	0.655	0.98 (0.74‒1.30)	0.904
Age, y	1.04 (1.01‒1.06)	0.002	1.00 (0.98‒1.01)	0.384
Body mass index, kg/m^2^	0.99 (0.92‒1.06)	0.694	1.00 (0.97‒1.04)	0.849
Hypertension	1.08 (0.66‒1.76)	0.761	1.00 (0.78‒1.27)	0.976
Prior myocardial infarction	0.82 (0.40‒1.69)	0.587	1.04 (0.72‒1.50)	0.856
History of cerebrovascular disease	1.94 (1.11‒3.40)	0.020	0.84 (0.58‒1.22)	0.357
Diabetes	1.55 (0.97‒2.45)	0.065	0.94 (0.74‒1.20)	0.631
Dyslipidemia	0.75 (0.46‒1.22)	0.245	1.03 (0.81‒1.31)	0.816
Chronic kidney disease	1.09 (0.51‒2.33)	0.832	0.96 (0.62‒1.49)	0.860
History of cancer	1.06 (0.48‒2.35)	0.879	0.99 (0.61‒1.60)	0.966
ST elevation on ECG	0.98 (0.51‒1.91)	0.957	0.99 (0.70‒1.41)	0.968
Abnormal Q wave on ECG	1.09 (0.70‒1.71)	0.700	0.99 (0.78‒1.25)	0.925
Right coronary artery	0.81 (0.51‒1.30)	0.390	1.04 (0.82‒1.32)	0.762
Atrial fibrillation	1.68 (0.88‒3.20)	0.116	0.86 (0.55‒1.35)	0.517
Killip class III or IV	1.42 (0.74‒2.70)	0.290	0.91 (0.60‒1.38)	0.645
Stenting performed	0.86 (0.51‒1.44)	0.556	1.03 (0.78‒1.35)	0.848
TIMI grade pre‐PCI	1.02 (0.83‒1.25)	0.852	0.99 (0.90‒1.10)	0.905
LVEF	1.01 (0.99‒1.03)	0.382	1.00 (0.99‒1.01)	0.648
LV aneurysm	2.79 (0.93‒8.37)	0.072	0.87 (0.42‒1.81)	0.715
IABP	1.68 (1.00‒2.82)	0.051	0.90 (0.66‒1.23)	0.512
Distal protection	1.41 (0.68‒2.91)	0.356	0.94 (0.61‒1.45)	0.789

Data are expressed as odds ratio with 95% CI. In the multilevel logistic regression model, each hospital was treated as a random effect. Results of the multivariable adjusted models are tabulated. IABP indicates intra‐aortic balloon pump; LV, left ventricular; LVEF, left ventricular ejection fraction; PCI, percutaneous coronary intervention; and TIMI, thrombolysis in myocardial infarction.

**Figure 4:**
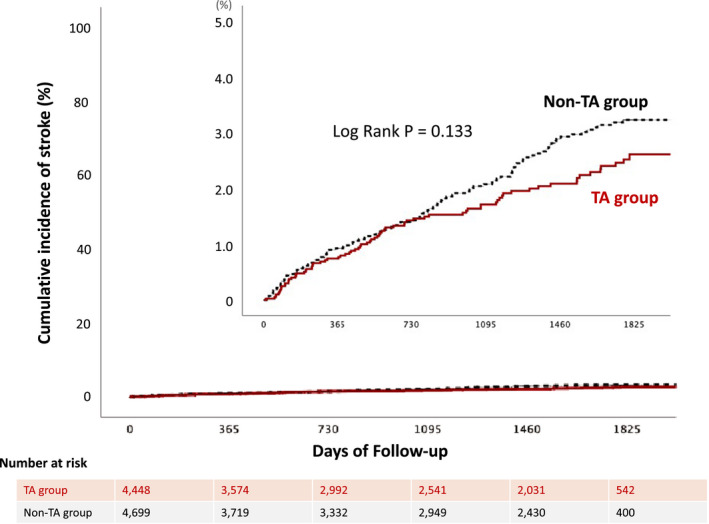
Kaplan–Meier estimates for stroke after discharge. Shown are the cumulative incidence of stroke after hospital discharge in the thrombus aspiration group (red line) and the non‐thrombus aspiration group (black dotted line) up to 5 years. The median follow‐up duration was 1805 days (interquartile range, 748‒1832). The inset shows a more detailed view of the same data up to a probability of 5.0%. Kaplan‒Meier analysis showed that there was no significant difference in the rate of stroke between both groups (Log rank, *P* =0.133). Main reason of the gradual decrease of number at risk was lost to follow‐up.

After discharge, stroke occurred in 78 patients (1.75%) in the TA group versus 107 patients (2.28%) in the non‐TA group, respectively (*P*=0.075). The median follow‐up duration was 1805 days (interquartile range, 748‒1832). Kaplan‒Meier analysis showed that there was no significant difference in the rate of stroke between both groups (Log rank, *P*=0.133) (Figure 4). Multivariable Cox proportional hazard model presented no significant impact of TA on stroke incidence after discharge (HR, 0.80 [95% CI, 0.58–1.11], *P*=0.182) (Table S8). The sensitivity analysis with Fine and Gray model also consistently showed the limited impact of TA on stroke events (HR, 0.83 [95% CI, 0.59–1.15], *P*=0.260). The E‐value to assess the potential effect of unmeasured confounding was 1.79.

## Discussion

The key findings of the present study are as follows: (1) manual TA during primary PCI for patients with AMI was independently associated with the increased risk of 7‐day stroke in the overall cohort; (2) this finding was consistently observed irrespective of high volume center or TA performance rate; (3) however, the observed incremental stroke risk was substantially driven by the results of some specific institutions; and (4) the multilevel random effects logistic regression models with hospital treated as a random effect presented the non‐incremental risk of 7‐day stroke by manual TA.

Not a small number of interventional cardiologists still perform TA, which may be because they feel some benefits from their clinical experiences. Previous small studies showed possible beneficial effects of TA: improved thrombolysis in myocardial infarction flow grade after PCI,^8^, ^9^, ^10^ prevention of microvascular obstruction,^10^ an efficient procedure with a reduced number of stents, short stent length and large stent size.^8^ These surrogate end points showed positive effects of manual TA in general. However, despite these supportive reports, the large landmark randomized trials failed to show the possible benefits of manual TA in terms of a composite end point of cardiovascular death, recurrent myocardial infarction, cardiogenic shock, or New York Heart Association class IV heart failure; or rather showed the increased risk of stroke in the TOTAL study.^4^, ^5^, ^6^, ^7^, ^13^ Based on these evidences, the current clinical guidelines grade routine TA during primary PCI as class III.^1^, ^2^, ^3^ The primary mechanism of stroke was thought to be embolization of thrombus or air to the brain during the procedure.^7^ Therefore, stroke associated with manual TA are thought to be closely related to insufficient procedural handling.^12^ A thrombus that cannot be fully aspirated is at risk of fracturing and shedding fragments into the systemic vasculature, particularly if fragments are sheared off as it is withdrawn into the guide catheter. A thrombus that cannot be fully aspirated through the catheter is at risk of exiting the aspiration catheter intact and entering the systemic vasculature, particularly if suction is not maintained on the aspiration catheter as it is withdrawn. In both cases, the risk may be increased if the guide catheter is not engaged in the artery when the aspiration catheter is withdrawn. Given these mechanisms, the excess risk of stroke should be observed in the periprocedural period. In this real‐world East Asian AMI registry, we detected a significant difference in the primary end point (stroke at 7 days) between the TA‐ and non‐TA groups. In the detailed analysis of stroke in the TOTAL study,^20^ the difference in stroke rates was apparent within 48 hours of primary PCI (15 [0.3%] versus 5 [0.1%], HR 3.00 [95% CI, 1.09–8.25]) but not at 12 hours after primary PCI (7 [0.14%] versus 4 [0.08%], HR 1.75 [95% CI, 0.51–5.98], *P*=0.37). Although we could not assess the incidences of 12 hours or 48 hours because of lack of data, our finding was consistent with that from TOTAL trial. Several meta‐analyses consistently reported the significant risk of stroke. Nevertheless, it was always driven by the results of the TOTAL study.^6^, ^13^, ^14^, ^15^ The other large‐scale trials, TASTE and TAPAS (The Thrombus Aspiration during Percutaneous Coronary Intervention in Acute Myocardial Infarction Study), did not show the excess of stroke events.^4^, ^5^, ^13^, ^21^

**Figure 5:**
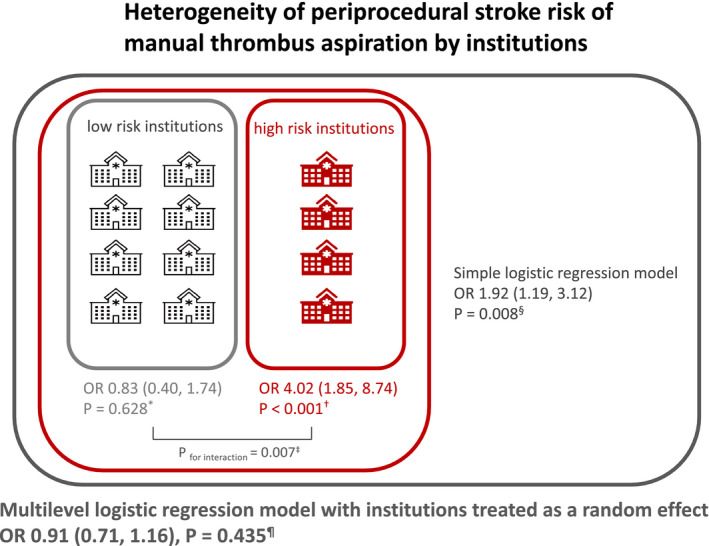
Heterogeneity of periprocedural stroke risk of manual thrombus aspiration by institutions. Manual TA during primary percutaneous coronary intervention for patients with acute myocardial infarction was independently associated with the overall increased risk of periprocedural stroke in the simple logistic regression model (odds ratio [OR], 1.92 [95% CI, 1.19‒3.12]; *P*=0.008^§^). However, this result was substantially skewed because of institution specific risk variation. In the low‐risk institutions, the incremental risk was not statistically significant (OR, 0.83 (95% CI, 0.40‒1.74), *P*=0.628^*^), whereas in the high‐risk institutions, manual TA was independently associated with periprocedural stroke (OR, 4.02 [95% CI, 1.85‒8.74]; *P*<0.001^†^) (*P*_for interaction_=0.007^‡^). The multilevel random effects logistic regression model with hospital treated as a random effect showed that manual TA was not associated with incremental risk of stroke at 7 days (OR, 0.91 [95% CI, 0.71‒1.16]; *P*=0.435^¶^). OR indicates odds ratio; and TA, thrombus aspiration.

In comparison with the findings from the simple logistic regression models, the random‐effects logistic regression models did not show the incremental periprocedural stroke risk of manual TA in this study. The harmful impact of manual TA was totally cancelled when considering hospitals treated as a random effect (Figure 5). Furthermore, our subgroup analysis showed the incremental risk of periprocedural stroke observed in the overall cohort was substantially driven by some specific institutions, which is however unexpectedly irrespective of high‐volume center or TA performance rate (Figure 3). This implies that the incremental risk of stroke was some specific operators‐dependent, which could not be assessed by the abovementioned parameters like TA performance rate and case number enrolled. Wide variety of medical standards in participating hospitals would also contribute to this finding. The periprocedural management in cath laboratories or cardiac care units is of paramount importance. It would be plausible that this might happen also in TOTAL study. High risk institutions and low risk institutions possibly coexisted also in TOTAL study. The overall finding may be driven by some specific high‐risk institutions. This would explain the inconsistent results in large‐scale trials including, TASTE, TAPAS, and TOTAL studies.^4^, ^5^, ^6^, ^7^, ^13^, ^20^, ^22^

On the contrary to the findings of TOTAL study, continuous effect of TA on long‐term stroke rates was not observed in the present study. Although unknown potential mechanisms influencing on the continuous risk of stroke could exist,^23^ it would be presumably a play of chance, given the relatively small number of events in the TOTAL study. Nonetheless, our findings should also be interpreted with caution because of the observational study design and long‐term enrollment period (1998–2014). We focused on stroke rather than the other efficacy outcomes in the present study. This is because it seems challenging to perform a scientifically sound analysis because of numerous confounders such as un‐prespecified PCI and medication strategy and very long enrollment period (1998–2014). During this long enrollment period, optimal medical therapy with angiotensin‐converting enzyme inhibitor/angiotensin II receptor blocker, beta blocker, and statin has been established; PCI with drug‐eluting stents rather than bare metal stents has become the main strategy for primary PCI.^24^ These drastic changes strongly affect the clinical outcomes including stroke at long‐term, myocardial infarction, revascularization, heart failure readmission, all‐cause death, and the composite end point of these outcomes. On the other hand, stroke, especially in the periprocedural period, should not be affected by these confounders. When assessing the impact of manual TA on 7‐day stroke, the impact of the confounders may be limited. Important differences in thrombogenicity and propensity for bleeding complications also exist between White and East Asian patients.^25^, ^26^ East Asian patients, in general, have a higher bleeding risk and a lower thrombogenic risk than White patients, which might have influenced on the long‐term results of this study. Generalizability of the current findings should be further tested in other populations.

### Clinical Implications

This study demonstrated that in the real‐world East Asian clinical practice, manual TA was not associated with the incremental risk of stroke. However, there was the significant heterogeneity of stroke risk by manual TA in participating hospitals (Figure 5). Clinical practice standards at the institutions participating in this registry, as well as the expertise of the interventional cardiologists who performed the procedures, may presumably differ by institutions. This point may have a strong message that the periprocedural stroke can be preventable by prudent procedure or appropriate periprocedural management. Even though the routine performance of manual TA does not provide beneficial clinical impact regarding cardiovascular death, recurrent myocardial infarction, and heart failure in the overall patients, efficient procedure with TA would be beneficial for physicians and patients in some cases.^8^ In order to prove the possible clinical efficacy suggested by the abovementioned positive results in surrogates, further operator‐oriented and patient‐selective large‐scale long‐term study would be warranted.

### Study Limitations

Several limitations should be acknowledged. First, we used an observational study design, rather than a randomized design, which would limit our findings hypothesis‐generating. As the impact of unmeasured confounding factors was presented by E‐value of 3.26, the robustness of the current findings may be partly confirmed. Second, the registry was performed in Japan, which might impair the generalizability of the results to the other countries and populations because of the racial differences and disparity of the clinical practice. Third, although it is unlikely that there was an underreporting of stroke events because independent clinical research coordinator systematically monitored clinical data of overall patients, event adjudication performed by on‐site investigators rather than an independent event adjudication committee might have resulted in underestimation or overestimation of the clinical events. Also, the definition of stroke might be outdated as compared with the most updated definition.^27^ Detailed classification of stroke (ischemic or hemorrhagic) was not available either. Fourth, PCI operators’ data was not available in our dataset, which does not allow us to investigate the relationship between physicians’ experience and periprocedural stroke risk. Finally, medical therapy might have influenced long‐term stroke rates. Although we statistically adjusted these confounders, unknown and unmeasured confounders cannot be adjusted enough, which is the inevitable limitation of the observational study. However, the impact of these potential confounders on “periprocedural” stroke events should be very limited as mentioned above.

## Conclusions

The present study from the large‐scale real‐world East Asian AMI registry showed that manual TA during primary PCI for patients with AMI was independently associated with the overall increased risk of periprocedural stroke. However, this result was substantially skewed because of institution specific risk variation, suggesting that the periprocedural stroke may be preventable by prudent PCI procedure or appropriate periprocedural management. Further operator‐oriented and patient‐selective clinical trials would be warranted to accurately investigate the clinical efficacy of manual TA.

## Sources of Funding

This work was supported by Grants‐in‐Aid for University and Society Collaboration (#19590816, #19390215, and #25461055) from the Japanese Ministry of Education, Culture, Sports, Science and Technology, Tokyo, Japan.

## Disclosures

Y. Sotomi received research grants from Abbott Medical Japan, and speaker honoraria from Abbott Medical Japan, Boston Scientific Japan, TERUMO, Japan Lifeline, Biosensors, and Medtronic and is an endowed chair funded by TERUMO, Asahi Intecc, NIPRO, and Shimadzu Corporation. Y. Ueda received research grants from Abbott Medical Japan and Medtronic, and lecture fees from NIPRO. H. Mizuno is an endowed chair funded by TERUMO, Asahi Intecc, NIPRO, and Shimadzu Corporation, and received personal fees from Medtronic Japan, Japan Lifeline, and Abbott Medical Japan. Y. Sakata received grants form Abbott Medical Japan and Biotronik. The remaining authors have no disclosures to report.

## Supporting information

Appendix
Figure S1–S3
Tables S1–S8

## References

[1] Levine GN , Bates ER , Blankenship JC , Bailey SR , Bittl JA , Cercek B , Chambers CE , Ellis SG , Guyton RA , Hollenberg SM , et al. 2015 ACC/AHA/SCAI focused update on primary percutaneous coronary intervention for patients with ST‐elevation myocardial infarction: an update of the 2011 ACCF/AHA/SCAI guideline for percutaneous coronary intervention and the 2013 ACCF/AHA guideline for the management of ST‐elevation myocardial infarction: a report of the American College of Cardiology/American Heart Association task force on clinical practice guidelines and the society for cardiovascular angiography and interventions. Circulation. 2016;133:1135–1147. DOI: 10.1161/CIR.0000000000000336. 26490017

[2] Neumann FJ , Sousa‐Uva M , Ahlsson A , Alfonso F , Banning AP , Benedetto U , Byrne RA , Collet JP , Falk V , Head SJ , et al. 2018 ESC/EACTS guidelines on myocardial revascularization. Eur Heart J. 2019;40:87–165. DOI: 10.1093/eurheartj/ehy394. 30165437

[3] Kimura K , Kimura T , Ishihara M , Nakagawa Y , Nakao K , Miyauchi K , Sakamoto T , Tsujita K , Hagiwara N , Miyazaki S , et al. JCS 2018 guideline on diagnosis and treatment of acute coronary syndrome. Circ J. 2019;83:1085–1196. DOI: 10.1253/circj.CJ-19-0133. 30930428

[4] Fröbert O , Lagerqvist BO , Olivecrona GK , Omerovic E , Gudnason T , Maeng M , Aasa M , Angerås O , Calais F , Danielewicz M , et al. Thrombus aspiration during ST‐segment elevation myocardial infarction. N Engl J Med. 2013;369:1587–1597. DOI: 10.1056/NEJMoa1308789. 23991656

[5] Lagerqvist BO , Fröbert O , Olivecrona GK , Gudnason T , Maeng M , Alström P , Andersson J , Calais F , Carlsson J , Collste O , et al. Outcomes 1 year after thrombus aspiration for myocardial infarction. N Engl J Med. 2014;371:1111–1120. DOI: 10.1056/NEJMoa1405707. 25176395

[6] Jolly SS , Cairns JA , Yusuf S , Rokoss MJ , Gao P , Meeks B , Kedev S , Stankovic G , Moreno R , Gershlick A , et al. Outcomes after thrombus aspiration for ST elevation myocardial infarction: 1‐year follow‐up of the prospective randomised total trial. Lancet. 2016;387:127–135. DOI: 10.1016/S0140-6736(15)00448-1. 26474811 PMC5007127

[7] Jolly SS , Cairns JA , Yusuf S , Meeks B , Pogue J , Rokoss MJ , Kedev S , Thabane L , Stankovic G , Moreno R , et al. Randomized trial of primary PCI with or without routine manual thrombectomy. N Engl J Med. 2015;372:1389–1398. DOI: 10.1056/NEJMoa1415098. 25853743 PMC4995102

[8] Fernandez‐Rodriguez D , Alvarez‐Contreras L , Martin‐Yuste V , Brugaletta S , Ferreira I , De Antonio M , Cardona M , Marti V , Garcia‐Picart J , Sabate M . Does manual thrombus aspiration help optimize stent implantation in ST‐segment elevation myocardial infarction? World J Cardiol. 2014;6:1030–1037. DOI: 10.4330/wjc.v6.i9.1030. 25276303 PMC4176794

[9] Gao L , Cao Z , Zhang H . Efficacy and safety of thrombectomy combined with intracoronary administration of tirofiban in ST‐segment elevation myocardial infarction (STEMI). Med Sci Monit. 2016;22:2699–2705. DOI: 10.12659/MSM.896703. 27475844 PMC4978207

[10] Sardella G , Mancone M , Bucciarelli‐Ducci C , Agati L , Scardala R , Carbone I , Francone M , Di Roma A , Benedetti G , Conti G , et al. Thrombus aspiration during primary percutaneous coronary intervention improves myocardial reperfusion and reduces infarct size: the expira (thrombectomy with export catheter in infarct‐related artery during primary percutaneous coronary intervention) prospective, randomized trial. J Am Coll Cardiol. 2009;53:309–315. DOI: 10.1016/j.jacc.2008.10.017. 19161878

[11] Nakatani D , Sato H , Sakata Y , Mizuno H , Shimizu M , Suna S , Nanto S , Hirayama A , Ito H , Fujii K , et al. Effect of intracoronary thrombectomy on 30‐day mortality in patients with acute myocardial infarction. Am J Cardiol. 2007;100:1212–1217. DOI: 10.1016/j.amjcard.2007.05.040. 17920359

[12] Brown ED , Blankenship JC . A mechanism for stroke complicating thrombus aspiration. Catheter Cardiovasc Interv. 2017;89:93–96. DOI: 10.1002/ccd.26682. 27696665

[13] Jolly SS , James S , Džavík V , Cairns JA , Mahmoud KD , Zijlstra F , Yusuf S , Olivecrona GK , Renlund H , Gao P , et al. Thrombus aspiration in ST‐segment‐elevation myocardial infarction: an individual patient meta‐analysis: thrombectomy trialists collaboration. Circulation. 2017;135:143–152. DOI: 10.1161/CIRCULATIONAHA.116.025371. 27941066

[14] Taglieri N , Bacchi Reggiani ML , Ghetti G , Saia F , Compagnone M , Lanati G , Di Dio MT , Bruno A , Bruno M , Della Riva D , et al. Efficacy and safety of thrombus aspiration in ST‐segment elevation myocardial infarction: an updated systematic review and meta‐analysis of randomised clinical trials. Eur Heart J Acute Cardiovasc Care. 2019;8:24–38. DOI: 10.1177/2048872618795512. 30160519

[15] Marmagkiolis K , Hakeem A , Cilingiroglu M , Feldman DN , Charitakis K . Efficacy and safety of routine aspiration thrombectomy during primary PCI for ST‐segment elevation myocardial infarction: a meta‐analysis of large randomized controlled trials. Hellenic J Cardiol. 2018;59:168–173. DOI: 10.1016/j.hjc.2017.09.003. 29241844

[16] Sotomi Y , Hikoso S , Nakatani D , Suna S , Dohi T , Mizuno H , Okada K , Kida H , Oeun B , Sunaga A , et al. Prevalence of the Japanese high bleeding risk criteria and its prognostic significance for fatal bleeding in patients with acute myocardial infarction. Heart Vessels. 2021. [epub ahead of print]. DOI: 10.1007/s00380-021-01836-9. 33743047

[17] Hara M , Sakata Y , Nakatani D , Suna S , Nishino M , Sato H , Kitamura T , Nanto S , Hori M , Komuro I , et al. Impact of coronary collaterals on in‐hospital and 5‐year mortality after ST‐elevation myocardial infarction in the contemporary percutaneous coronary intervention era: a prospective observational study. BMJ Open. 2016;6:e011105. DOI: 10.1136/bmjopen-2016-011105. PMC4947770 27412101

[18] Fine JP , Gray RJ . A proportional hazards model for the subdistribution of a competing risk. J Am Stat Assoc. 1999;94:496–509. DOI: 10.1080/01621459.1999.10474144.

[19] VanderWeele TJ , Ding P . Sensitivity analysis in observational research: introducing the E‐value. Ann Intern Med. 2017;167:268–274. DOI: 10.7326/M16-2607. 28693043

[20] Jolly SS , Cairns JA , Yusuf S , Meeks B , Gao P , Hart RG , Kedev S , Stankovic G , Moreno R , Horak D , et al. Stroke in the total trial: a randomized trial of routine thrombectomy vs. percutaneous coronary intervention alone in ST elevation myocardial infarction. Eur Heart J. 2015;36:2364–2372. DOI: 10.1093/eurheartj/ehv296. 26129947 PMC4568405

[21] Svilaas T , Vlaar PJ , van der Horst IC , Diercks GFH , de Smet BJGL , van den Heuvel AFM , Anthonio RL , Jessurun GA , Tan ES , Suurmeijer AJH , et al. Thrombus aspiration during primary percutaneous coronary intervention. N Engl J Med. 2008;358:557–567. DOI: 10.1056/NEJMoa0706416. 18256391

[22] Vlaar PJ , Svilaas T , van der Horst IC , Diercks GFH , Fokkema ML , de Smet BJGL , van den Heuvel AFM , Anthonio RL , Jessurun GA , Tan ES , et al. Cardiac death and reinfarction after 1 year in the thrombus aspiration during percutaneous coronary intervention in acute myocardial infarction study (TAPAS): a 1‐year follow‐up study. Lancet. 2008;371:1915–1920. DOI: 10.1016/S0140-6736(08)60833-8. 18539223

[23] Dutta P , Courties G , Wei Y , Leuschner F , Gorbatov R , Robbins CS , Iwamoto Y , Thompson B , Carlson AL , Heidt T , et al. Myocardial infarction accelerates atherosclerosis. Nature. 2012;487:325–329. DOI: 10.1038/nature11260. 22763456 PMC3401326

[24] Sotomi Y , Suzuki S , Kobayashi T , Hamanaka Y , Nakatani S , Hirata A , Takeda Y , Ueda Y , Sakata Y , Higuchi Y . Impact of the one‐year angioscopic findings on long‐term clinical events in 504 patients treated with first‐generation or second‐generation drug‐eluting stents: the DESNOTE‐X study. EuroIntervention. 2019;15:631–639. DOI: 10.4244/EIJ-D-18-00660. 30398964

[25] Levine GN , Jeong YH , Goto S , Anderson JL , Huo Y , Mega JL , Taubert K , Smith SC Jr . Expert consensus document: World Heart Federation expert consensus statement on antiplatelet therapy in East Asian patients with ACS or undergoing PCI. Nat Rev Cardiol. 2014;11:597–606. DOI: 10.1038/nrcardio.2014.104. 25154978

[26] Sotomi Y , Onuma Y , Cavalcante R , Ahn J‐M , Lee CW , van Klaveren D , de Winter RJ , Wykrzykowska JJ , Farooq V , Morice M‐C , et al. Geographical difference of the interaction of sex with treatment strategy in patients with multivessel disease and left main disease: a meta‐analysis from syntax (synergy between PCI with taxus and cardiac surgery), precombat (bypass surgery versus angioplasty using sirolimus‐eluting stent in patients with left main coronary artery disease), and best (bypass surgery and everolimus‐eluting stent implantation in the treatment of patients with multivessel coronary artery disease) randomized controlled trials. Circ Cardiovasc Interv. 2017;10. DOI: 10.1161/CIRCINTERVENTIONS.117.005027. 28495897

[27] Sacco RL , Kasner SE , Broderick JP , Caplan LR , Connors JJ , Culebras A , Elkind MSV , George MG , Hamdan AD , Higashida RT , et al. An updated definition of stroke for the 21st century: a statement for healthcare professionals from the American Heart Association/American Stroke Association. Stroke. 2013;44:2064–2089. DOI: 10.1161/STR.0b013e318296aeca. 23652265 PMC11078537

