# Supplementary material for: Manual Thrombus Aspiration and its Procedural Stroke Risk in Myocardial Infarction
Source: J Am Heart Assoc. 2021 Nov 15;10(22):e022258. doi: 10.1161/JAHA.121.022258 (PMC8751963; doi:10.1161/JAHA.121.022258)
Supplement: Supplementary file 1 — Appendix Figure S1–S3 Tables S1–S8 [file JAH3-10-e022258-s001.pdf]

SUPPLEMENTAL MATERIAL

The Osaka Acute Coronary Insufficiency Study (OACIS) Group

Osaka University Graduate School of Medicine, Suita, Japan; Higashi-Osaka Medical Center, Higashi-Osaka, Japan; Kawachi General Hospital, Higashi-Osaka, Japan; Kwasnsei Gakuin University, Nishinomiya, Japan; Kobe Ekisaikai Hospital, Kobe, Japan; Kansai Rosai Hospital, Amagasaki, Japan; Meiwa Hospital, Nishinomiya, Japan; Osaka General Medical Center, Osaka, Japan; Osaka Hospital, Japan Community Healthcare Organization Osaka, Japan; Osaka Minami Medical Center, National Hospital Organization, Kawachinagano, Japan; Osaka Medical Center, National Hospital Organization, Osaka, Japan; Osaka International Cancer Institute, Osaka, Japan; Osaka Police Hospital, Osaka, Japan; Osaka Rosai Hospital, Sakai, Japan; Osaka Seamens Insurance Hospital, Osaka, Japan; Saiseikai Senri Hospital, Suita, Japan; Sakurabashi Watanabe Hospital, Osaka, Japan; Settsu Iseikai Hospital, Settsu, Japan; Teramoto Memorial Hospital, Kawachinagano, Japan; and Yao Municipal Hospital, Yao, Japan.

Figure S1. Serial change of TA performance rate.

Patients were enrolled from 1998 to 2014. Serial change of TA performance rate and incidence of periprocedural stroke (at 7 days) are illustrated as line charts. Bar charts indicate annual number of patient enrollment. TA performance rate and periprocedural stroke incidence gradually increased in the study period ($P_{for\ trend} < 0.001$, $P_{for\ trend} = 0.031$, respectively; Cochran-Armitage trend test). Abbreviations: TA, thrombus aspiration.

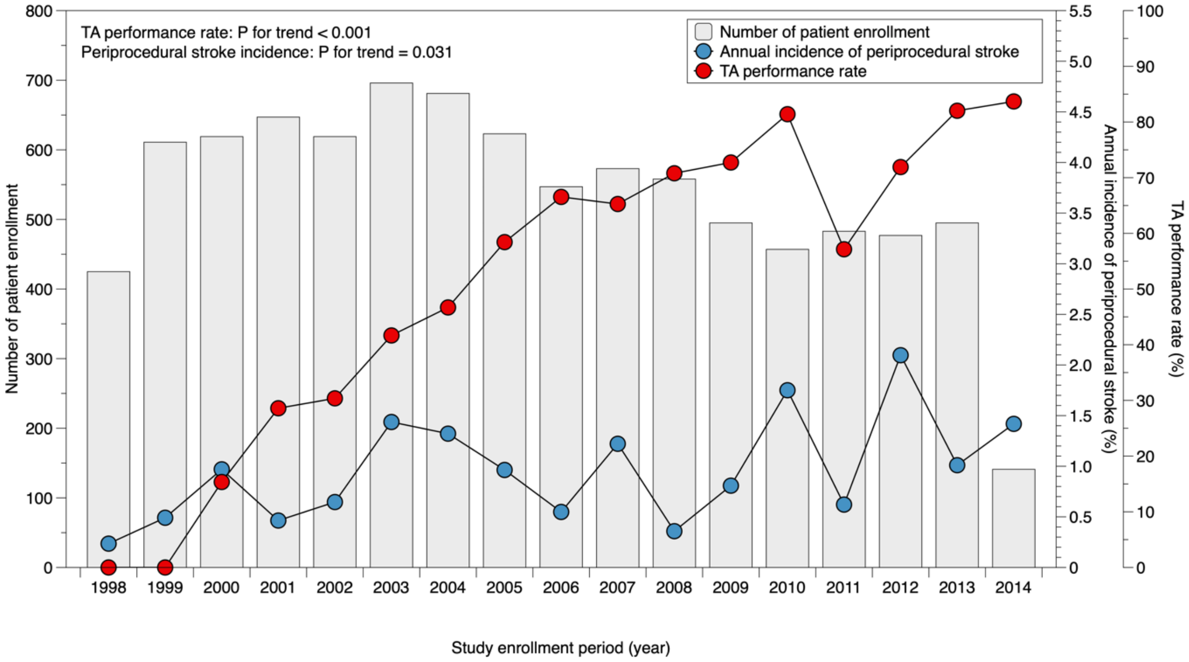

Figure S2. Scatter plots of 7-day stroke incidence and thrombus aspiration performance rate by institutions.

Shown are scatter plots of 7-day stroke incidence by institutions. Each plot shows each institution. Horizontal axis indicates the performance rate of thrombus aspiration. Vertical axis shows the incidence of the primary endpoint (stroke at 7 days). No significant relationship was found between thrombus aspiration performance rate and incidence of stroke at 7 days.

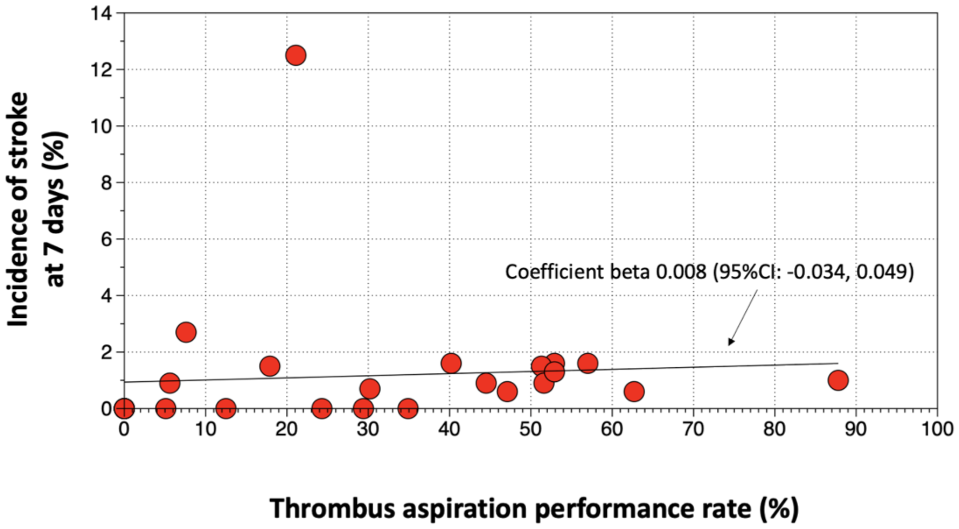

Figure S3. Scatter plots of 7-day stroke incidence and procedural volume by institutions.

Shown are scatter plots of 7-day stroke incidence by institutions. Each plot shows each institution. Horizontal axis indicates sample size in each institution. Vertical axis shows the incidence of the primary endpoint (stroke at 7 days). No significant relationship was found between procedural volume and incidence of stroke at 7 days.

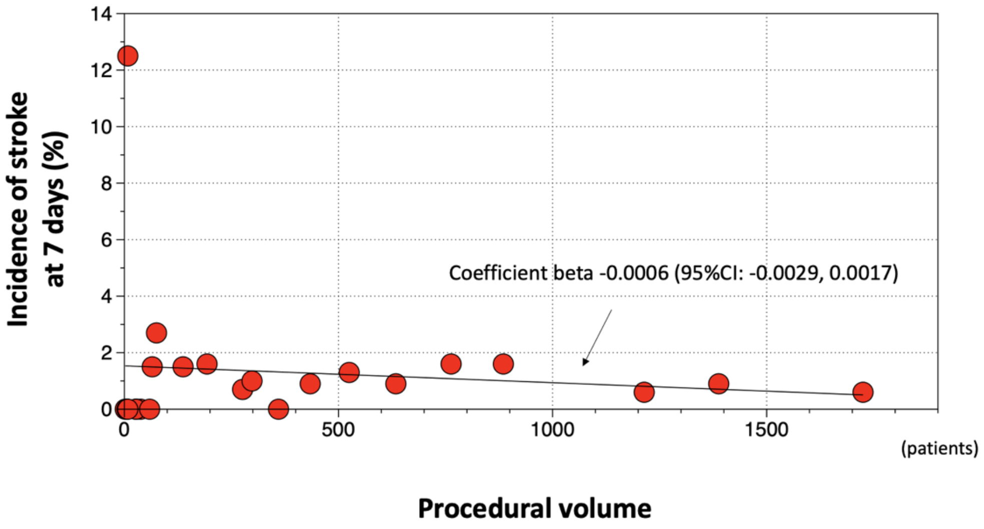

Table S1. Patient characteristics (included vs. excluded from the analysis)

	Patients excluded	Patients analysed	P value	Missing (%)
Patients, n	2946	9147		
Age, years	68.0 [59.0, 77.0]	66.0 [58.0, 75.0]	<0.001	0
Male sex	2116 (71.8)	6980 (76.3)	<0.001	0
Diabetes mellitus	986 (35.3)	3023 (34.0)	0.191	3.3
Hypertension	1656 (59.9)	5486 (61.8)	0.079	3.7
Dyslipidaemia	1103 (40.5)	3914 (44.7)	<0.001	5.1
Smoking	1643 (59.3)	5729 (64.2)	<0.001	3.3
Chronic kidney disease	278 (9.9)	628 (7.1)	<0.001	3.6
Atrial fibrillation	247 (8.8)	535 (6.0)	<0.001	2.7
Prior myocardial infarction	365 (12.9)	1035 (11.6)	0.056	2.8
History of cerebrovascular disease	332 (11.8)	808 (9.1)	<0.001	3.6
History of cancer	180 (6.4)	517 (5.8)	0.305	3.6

	Patients excluded	Patients analysed	P value	Missing (%)
Systolic blood pressure on admission, mmHg	130.0 [109.0, 152.0]	136.0 [117.0, 156.0]	<0.001	33
Diastolic blood pressure on admission, mmHg	78.0 [64.0, 90.0]	80.0 [68.0, 92.0]	<0.001	34.7
Heart rate on admission, bpm	80.0 [67.0, 97.0]	78.0 [65.0, 91.0]	<0.001	33
Low density lipoprotein cholesterol, mg/dL	107.60 [83.0, 136.40]	121.0 [97.0, 146.0]	<0.001	63.9
HbA1c, %	5.60 [5.10, 6.50]	5.60 [5.20, 6.50]	0.048	27.2
Killip class III or IV	646 (21.9)	770 (8.4)	<0.001	0
ST elevation on ECG	2185 (77.6)	7865 (87.2)	<0.001	2.1
Abnormal Q wave on ECG	1374 (49.4)	4060 (46.0)	0.002	4
Time from symptom onset to primary PCI, hour	5.0 [2.50, 15.50]	4.0 [2.0, 11.0]	<0.001	19.7
Culprit vessel				
Right coronary artery	692 (31.5)	3212 (36.4)	<0.001	8.9
Left main trunk or left anterior descending artery	1152 (52.4)	4378 (49.7)	0.023	8.9
Left circumflex artery	377 (17.1)	1357 (15.4)	0.047	8.9
TIMI grade pre PCI			<0.001	20.9

	Patients excluded	Patients analysed	P value	Missing (%)
TIMI 0	834 (44.7)	4239 (55.0)		
TIMI 1	193 (10.4)	865 (11.2)		
TIMI 2	306 (16.4)	1488 (19.3)		
TIMI 3	531 (28.5)	1114 (14.5)		
Collateral blood flow (+)	650 (29.9)	2976 (33.3)	0.003	8.1
Stenting performed	868 (29.5)	6346 (69.4)	<0.001	0
Post PCI laboratory data				
Peak CK, IU/L	1642.50 [744.25, 3888.25]	2025.0 [956.0, 3800.0]	<0.001	6
Peak CK-MB, IU/L	141.70 [63.0, 339.0]	178.0 [85.0, 339.0]	<0.001	14
Medication at discharge				
ACEi or ARB	1474 (65.3)	6708 (77.5)	<0.001	9.7
Beta blocker	991 (43.9)	4925 (56.9)	<0.001	9.7
Statin	847 (37.5)	4489 (51.8)	<0.001	9.7
Antiplatelets	2142 (94.8)	8510 (98.3)	<0.001	9.7

	Patients excluded	Patients analysed	P value	Missing (%)
Anticoagulants	567 (25.1)	1369 (15.8)	<0.001	9.7
LVEF	50.86 [40.47, 60.07]	53.85 [45.21, 61.03]	<0.001	31.7
LV thrombus	28 (1.6)	79 (1.2)	0.217	31.8
LV aneurysm	45 (2.6)	131 (2.0)	0.158	32.5
Length of hospitalization, days	22.0 [12.0, 34.0]	20.0 [14.0, 29.0]	0.028	0
Enrolment period (year)			<0.001	0
1998–2003	1607 (54.5)	3617 (39.5)		
2004–2009	837 (28.4)	3477 (38.0)		
2010–2014	502 (17.0)	2053 (22.4)		

Data are expressed as median [interquartile range] or number (percentage). Abbreviations: TA, thrombus aspiration; ECG, electrocardiogram; CAG, coronary angiography; TIMI, thrombolysis in myocardial infarction; PCI, percutaneous coronary intervention; CK, creatine kinase; CK-MB, creatine kinase myocardial band; ACEi, angiotensin converting enzyme inhibitor; ARB, angiotensin II receptor blocker, LVEF, left ventricular ejection fraction; LV, left ventricular.

Table S2. Impact of thrombus aspiration on the secondary endpoint of stroke from day 7 to hospital discharge.

	Simple logistic regression model		Multilevel logistic regression model	
	Stroke from day 7 to hospital discharge		Stroke from day 7 to hospital discharge	
	OR (95%CI)	P value	OR (95%CI)	P value
Thrombus aspiration	1.27 (0.66, 2.44)	0.468	0.98 (0.76, 1.27)	0.876
Male sex	1.20 (0.59, 2.44)	0.609	0.99 (0.74, 1.32)	0.920
Age, year	1.03 (1.00, 1.07)	0.038	1.00 (0.99, 1.01)	0.683
Body mass index, kg/m2	1.00 (0.92, 1.09)	0.987	1.00 (0.97, 1.04)	0.974
Hypertension	1.88 (0.90, 3.90)	0.092	0.96 (0.74, 1.24)	0.731
Prior myocardial infarction	0.86 (0.35, 2.13)	0.746	1.02 (0.70, 1.49)	0.914
History of cerebrovascular disease	1.25 (0.54, 2.88)	0.604	0.97 (0.64, 1.46)	0.889
Diabetes mellitus	0.75 (0.38, 1.48)	0.404	1.03 (0.80, 1.33)	0.831
Dyslipidemia	0.89 (0.46, 1.70)	0.724	1.01 (0.79, 1.29)	0.934
Chronic kidney disease	1.34 (0.51, 3.51)	0.558	0.97 (0.61, 1.54)	0.901
History of cancer	1.31 (0.46, 3.74)	0.621	0.97 (0.59, 1.61)	0.917

ST elevation on ECG	1.06 (0.43, 2.65)	0.899	1.00 (0.70, 1.43)	0.987
Abnormal Q wave on ECG	0.60 (0.32, 1.15)	0.123	1.04 (0.82, 1.33)	0.746
Right coronary artery	1.02 (0.54, 1.95)	0.944	1.00 (0.78, 1.28)	0.994
Atrial fibrillation	2.02 (0.89, 4.58)	0.093	0.88 (0.55, 1.42)	0.608
Killip class III or IV	2.02 (0.91, 4.49)	0.084	0.89 (0.58, 1.38)	0.603
Stenting performed	0.59 (0.30, 1.13)	0.111	1.05 (0.79, 1.38)	0.745
TIMI grade pre PCI	0.90 (0.67, 1.21)	0.476	1.01 (0.91, 1.12)	0.835
LVEF	0.99 (0.96, 1.01)	0.276	1.00 (0.99, 1.01)	0.775
LV aneurysm	1.91 (0.44, 8.35)	0.392	0.95 (0.43, 2.08)	0.893
IABP	1.75 (0.87, 3.52)	0.115	0.94 (0.68, 1.30)	0.712
Distal protection	0.69 (0.16, 2.95)	0.614	1.02 (0.64, 1.62)	0.933

Data are expressed odds ratio (OR) with 95% confidence interval (CI). In the multilevel logistic regression model, each hospital was treated as a random effect. Results of the multivariable adjusted models are tabulated. Abbreviations: ECG, electrocardiogram; TIMI, thrombolysis in myocardial infarction; IABP, intra-aortic balloon pump; LVEF, left ventricular ejection fraction; LV, left ventricular.

Table S3. Impact of thrombus aspiration on the secondary endpoint of in-hospital stroke.

	**Simple logistic regression model**		**Multilevel logistic regression model**	
	**in-hospital stroke**		**in-hospital stroke**	
	OR (95%CI)	P value	OR (95%CI)	P value
Thrombus aspiration	1.46 (0.96, 2.21)	0.075	0.93 (0.73, 1.19)	0.561
Male sex	1.03 (0.66, 1.59)	0.912	1.00 (0.76, 1.31)	0.994
Age, year	1.03 (1.01, 1.05)	0.002	1.00 (0.98, 1.01)	0.335
Body mass index, kg/m2	0.98 (0.92, 1.04)	0.512	1.01 (0.97, 1.04)	0.780
Hypertension	1.39 (0.89, 2.16)	0.149	0.95 (0.74, 1.21)	0.663
Prior myocardial infarction	0.82 (0.45, 1.51)	0.527	1.05 (0.73, 1.50)	0.793
History of cerebrovascular disease	1.74 (1.06, 2.87)	0.030	0.83 (0.58, 1.20)	0.320
Diabetes mellitus	1.25 (0.83, 1.89)	0.280	0.97 (0.76, 1.23)	0.780
Dyslipidemia	0.76 (0.50, 1.18)	0.221	1.04 (0.82, 1.31)	0.751
Chronic kidney disease	1.01 (0.51, 1.98)	0.984	0.98 (0.64, 1.50)	0.924
History of cancer	0.96 (0.46, 2.00)	0.902	1.02 (0.63, 1.63)	0.943

ST elevation on ECG	0.92 (0.52, 1.62)	0.772	1.01 (0.72, 1.42)	0.954
Abnormal Q wave on ECG	0.87 (0.59, 1.30)	0.499	1.03 (0.82, 1.29)	0.803
Right coronary artery	0.84 (0.55, 1.27)	0.402	1.04 (0.82, 1.32)	0.740
Atrial fibrillation	1.70 (0.97, 2.99)	0.065	0.83 (0.54, 1.27)	0.386
Killip class III or IV	1.80 (1.05, 3.06)	0.031	0.81 (0.55, 1.20)	0.286
Stenting performed	0.83 (0.53, 1.29)	0.410	1.04 (0.80, 1.35)	0.784
TIMI grade pre PCI	1.00 (0.83, 1.20)	0.978	1.00 (0.91, 1.10)	0.989
LVEF	1.00 (0.98, 1.02)	0.710	1.00 (0.99, 1.01)	0.956
LV aneurysm	2.09 (0.72, 6.07)	0.181	0.94 (0.46, 1.95)	0.871
IABP	1.54 (0.98, 2.44)	0.063	0.89 (0.66, 1.21)	0.463
Distal protection	1.30 (0.66, 2.58)	0.451	0.95 (0.62, 1.45)	0.807

Data are expressed odds ratio (OR) with 95% confidence interval (CI). In the multilevel logistic regression model, each hospital was treated as a random effect. Results of the multivariable adjusted models are tabulated. Abbreviations: ECG, electrocardiogram; TIMI, thrombolysis in myocardial infarction; IABP, intra-aortic balloon pump; LVEF, left ventricular ejection fraction; LV, left ventricular.

Table S4. Impact of thrombus aspiration on the primary endpoint (Minimum model)

	**Simple logistic regression model**		**Multilevel logistic regression model**	
	**Stroke at 7 days**		**Stroke at 7 days**	
	OR (95%CI)	P value	OR (95%CI)	P value
Thrombus aspiration	1.81 (1.17, 2.82)	0.008	0.91 (0.73, 1.15)	0.434
Male sex	1.12 (0.68, 1.85)	0.661	0.99 (0.75, 1.30)	0.921
Age, year	1.04 (1.02, 1.06)	0.000	1.00 (0.98, 1.01)	0.335
Hypertension	0.99 (0.62, 1.58)	0.968	1.00 (0.79, 1.28)	0.975
History of cerebrovascular disease	2.04 (1.19, 3.50)	0.010	0.84 (0.58, 1.22)	0.359
Diabetes mellitus	1.43 (0.92, 2.21)	0.113	0.95 (0.75, 1.21)	0.674
History of cancer	1.08 (0.49, 2.38)	0.846	0.99 (0.61, 1.59)	0.949
Atrial fibrillation	1.74 (0.92, 3.29)	0.086	0.86 (0.55, 1.34)	0.507
IABP	1.65 (1.00, 2.73)	0.052	0.91 (0.67, 1.23)	0.538
Killip class III or IV	1.38 (0.74, 2.58)	0.309	0.92 (0.61, 1.38)	0.676

Data are expressed odds ratio (OR) with 95% confidence interval (CI). In the multilevel logistic regression model, each hospital was treated as a random effect. Results of the multivariable adjusted models are tabulated. Abbreviations: IABP, intra-aortic balloon pump.

Table S5. Impact of thrombus aspiration on the secondary endpoint of stroke from day 7 to hospital discharge (Minimum model)

	Simple logistic regression model		Multilevel logistic regression model	
	Stroke from day 7 to hospital discharge		Stroke from day 7 to hospital discharge	
	OR (95%CI)	P value	OR (95%CI)	P value
Thrombus aspiration	1.07 (0.59, 1.95)	0.819	0.99 (0.79, 1.26)	0.964
Male sex	1.20 (0.60, 2.42)	0.606	0.99 (0.74, 1.32)	0.926
Age, year	1.04 (1.01, 1.07)	0.024	1.00 (0.99, 1.01)	0.662
Hypertension	1.79 (0.87, 3.68)	0.112	0.96 (0.75, 1.23)	0.753
History of cerebrovascular disease	1.27 (0.56, 2.92)	0.569	0.97 (0.64, 1.46)	0.882
Diabetes mellitus	0.73 (0.38, 1.42)	0.354	1.03 (0.80, 1.32)	0.828
History of cancer	1.27 (0.45, 3.62)	0.652	0.97 (0.59, 1.61)	0.919
Atrial fibrillation	2.24 (1.01, 4.99)	0.048	0.87 (0.55, 1.40)	0.569
IABP	1.94 (0.98, 3.83)	0.056	0.93 (0.68, 1.28)	0.664
Killip class III or IV	2.09 (0.96, 4.54)	0.063	0.89 (0.58, 1.36)	0.583

Data are expressed odds ratio (OR) with 95% confidence interval (CI). In the multilevel logistic regression model, each hospital was treated as a random effect. Results of the multivariable adjusted models are tabulated. Abbreviations: IABP, intra-aortic balloon pump.

Table S6. Impact of thrombus aspiration on the secondary endpoint of in-hospital stroke (Minimum model)

	**Simple logistic regression model**		**Multilevel logistic regression model**	
	**in-hospital stroke**		**in-hospital stroke**	
	OR (95%CI)	P value	OR (95%CI)	P value
Thrombus aspiration	1.37 (0.94, 2.01)	0.103	0.94 (0.75, 1.18)	0.588
Male sex	1.04 (0.67, 1.60)	0.872	1.00 (0.76, 1.31)	0.998
Age, year	1.04 (1.02, 1.06)	0.000	0.99 (0.98, 1.00)	0.263
Hypertension	1.27 (0.83, 1.95)	0.266	0.96 (0.76, 1.22)	0.741
History of cerebrovascular disease	1.81 (1.11, 2.95)	0.017	0.83 (0.58, 1.20)	0.321
Diabetes mellitus	1.17 (0.79, 1.73)	0.433	0.98 (0.77, 1.23)	0.831
History of cancer	0.97 (0.47, 2.02)	0.938	1.01 (0.63, 1.62)	0.967
Atrial fibrillation	1.77 (1.02, 3.09)	0.043	0.82 (0.54, 1.26)	0.361
IABP	1.61 (1.03, 2.51)	0.036	0.89 (0.66, 1.20)	0.446
Killip class III or IV	1.86 (1.11, 3.12)	0.018	0.81 (0.55, 1.18)	0.272

Data are expressed odds ratio (OR) with 95% confidence interval (CI). In the multilevel logistic regression model, each hospital was treated as a random effect. Results of the multivariable adjusted models are indicated. Abbreviations: IABP, intra-aortic balloon pump.

Table S7. Patient characteristics (high- vs. low-risk institutions)

	Institutions with high periprocedural stroke risk	Institutions with low periprocedural stroke risk	P value	Missing (%)
Patients, n	5058	4089		
Age, years	66.0 [58.0, 74.0]	67.0 [59.0, 75.0]	0.003	0
Male sex	3875 (76.6)	3105 (75.9)	0.454	0
Diabetes mellitus	1699 (34.3)	1324 (33.5)	0.403	2.7
Hypertension	3085 (62.7)	2401 (60.7)	0.064	3
Dyslipidaemia	2218 (45.6)	1696 (43.5)	0.048	4.2
Smoking	3096 (62.8)	2633 (65.8)	0.003	2.4
Chronic kidney disease	310 (6.3)	318 (8.1)	0.001	3.4
Atrial fibrillation	300 (6.0)	235 (5.9)	0.758	2
Prior myocardial infarction	632 (12.9)	403 (10.0)	<0.001	2.3
History of cerebrovascular disease	428 (8.7)	380 (9.7)	0.121	3.4
History of cancer	267 (5.4)	250 (6.4)	0.068	3.4

Systolic blood pressure on admission, mmHg	136.0 [117.0, 156.0]	137.0 [116.0, 156.0]	0.606	29.3
Diastolic blood pressure on admission, mmHg	80.0 [69.0, 91.0]	80.0 [68.0, 94.0]	0.236	30.8
Heart rate on admission, bpm	78.0 [64.0, 90.0]	78.0 [65.0, 92.0]	0.614	29.5
Low density lipoprotein cholesterol, mg/dL	121.90 [98.0, 148.0]	120.0 [96.0, 144.0]	0.093	60.3
HbA1c, %	5.60 [5.20, 6.50]	5.60 [5.20, 6.50]	0.158	24.2
Killip class III or IV	387 (7.7)	383 (9.4)	0.004	0
ST elevation on ECG	4306 (86.6)	3559 (87.9)	0.074	1.4
Abnormal Q wave on ECG	1924 (40.1)	2136 (53.0)	<0.001	3.5
Time from symptom onset to primary PCI, hour	4.00 [2.30, 11.40]	4.00 [2.00, 10.50]	0.002	13.6
Culprit vessel				
Right coronary artery	1793 (36.7)	1419 (36.2)	0.644	3.6
Left main trunk or left anterior descending artery	2421 (49.5)	1957 (49.9)	0.745	3.6
Left circumflex artery	791 (16.2)	566 (14.4)	0.026	3.6
TIMI grade pre PCI			0.004	15.8
TIMI 0	2408 (55.2)	1831 (54.8)		
TIMI 1	526 (12.1)	339 (10.1)		

TIMI 2	845 (19.4)	643 (19.2)		
TIMI 3	586 (13.4)	528 (15.8)		
Collateral blood flow (+)	1905 (38.6)	1071 (26.7)	<0.001	2.2
Stenting performed	3580 (70.8)	2766 (67.6)	0.001	0
Post PCI laboratory data				
Peak CK, IU/L	2018.0 [927.0, 3712.0]	2039.0 [995.0, 3881.75]	0.087	4.2
Peak CK-MB, IU/L	175.0 [83.0, 333.08]	183.0 [88.0, 348.28]	0.008	10.8
Medication at discharge				
ACEi or ARB	3765 (78.2)	2943 (76.6)	0.072	5.3
Beta blocker	2783 (57.8)	2142 (55.7)	0.054	5.3
Statin	2279 (47.3)	2210 (57.5)	<0.001	5.3
Antiplatelets	4740 (98.5)	3770 (98.1)	0.194	5.3
Anticoagulants	683 (14.2)	686 (17.8)	<0.001	5.3
LVEF	53.60 [44.74, 60.56]	54.74 [45.66, 61.65]	<0.001	27.9
LV thrombus	44 (1.2)	35 (1.2)	>0.999	28.6
LV aneurysm	73 (2.0)	58 (2.1)	0.902	29.5

Length of hospitalization, days	21.0 [14.0, 28.0]	19.0 [14.0, 29.0]	0.409	0
Enrolment period (year)			<0.001	0
1998–2003	2052 (40.6)	1565 (38.3)		
2004–2009	2020 (39.9)	1457 (35.6)		
2010–2014	986 (19.5)	1067 (26.1)		

Data are expressed as median [interquartile range] or number (percentage). Abbreviations: TA, thrombus aspiration; ECG, electrocardiogram; CAG, coronary angiography; TIMI, thrombolysis in myocardial infarction; PCI, percutaneous coronary intervention; CK, creatine kinase; CK-MB, creatine kinase myocardial band; ACEi, angiotensin converting enzyme inhibitor; ARB, angiotensin II receptor blocker, LVEF, left ventricular ejection fraction; LV, left ventricular.

Table S8. Impact of thrombus aspiration on long-term stroke event

	Cox proportional hazard model		Fine and Gray model	
	HR (95% CI)	P value	Subdistributional HR (95% CI)	P value
Thrombus aspiration	0.80 (0.58, 1.11)	0.182	0.83 (0.59, 1.15)	0.260
Male sex	0.96 (0.68, 1.36)	0.805	0.97 (0.69, 1.38)	0.880
Age, year	1.02 (1.01, 1.04)	0.004	1.02 (1.00, 1.03)	0.016
Body mass index, kg/m^2	0.98 (0.94, 1.02)	0.373	0.99 (0.95, 1.03)	0.520
Hypertension	1.81 (1.27, 2.59)	0.001	1.80 (1.26, 2.57)	0.001
Prior myocardial infarction	1.48 (0.99, 2.21)	0.054	1.48 (0.98, 2.22)	0.064
History of cerebrovascular disease	1.93 (1.31, 2.86)	0.001	1.84 (1.23, 2.75)	0.003
Diabetes mellitus	1.40 (1.04, 1.89)	0.029	1.37 (1.01, 1.86)	0.042
Dyslipidemia	0.71 (0.52, 0.97)	0.031	0.74 (0.54, 1.02)	0.066
Chronic kidney disease	1.29 (0.77, 2.15)	0.339	1.13 (0.66, 1.94)	0.650
History of cancer	1.97 (1.24, 3.13)	0.004	1.85 (1.16, 2.94)	0.010
ST elevation on ECG	0.78 (0.52, 1.16)	0.210	0.77 (0.51, 1.15)	0.190

	Cox proportional hazard model		Fine and Gray model	
	**HR (95% CI)**	**P value**	**Subdistributional HR (95% CI)**	**P value**
Abnormal Q wave on ECG	1.21 (0.90, 1.62)	0.215	1.20 (0.90, 1.60)	0.220
Right coronary artery	1.20 (0.89, 1.63)	0.239	1.20 (0.89, 1.63)	0.230
Atrial fibrillation	2.17 (1.40, 3.37)	0.001	2.08 (1.31, 3.30)	0.002
Killip class III or IV	0.98 (0.57, 1.70)	0.941	0.69 (0.40, 1.19)	0.180
Stenting performed	0.73 (0.53, 1.02)	0.064	0.74 (0.53, 1.02)	0.064
TIMI grade pre PCI	1.11 (0.98, 1.26)	0.098	1.11 (0.99, 1.26)	0.081
LVEF	1.00 (0.99, 1.02)	0.539	1.01 (1.00, 1.02)	0.190
LV aneurysm	0.85 (0.31, 2.33)	0.749	0.81 (0.28, 2.36)	0.700
LV thrombus	1.38 (0.43, 4.41)	0.583	1.32 (0.41, 4.30)	0.640
IABP	1.60 (1.10, 2.30)	0.013	1.42 (0.99, 2.03)	0.054
Distal protection	1.49 (0.88, 2.54)	0.140	1.55 (0.91, 2.64)	0.110
ACEi or ARB	1.20 (0.83, 1.74)	0.336	1.16 (0.80, 1.69)	0.430
Beta blocker	1.48 (1.07, 2.03)	0.016	1.60 (1.17, 2.18)	0.003

Data are expressed hazard ratio (HR) with 95% confidence interval (CI). We performed the main analysis with Cox proportional hazard model and the sensitivity analysis with a Fine–Gray model to account for the competing risk of all-cause death from stroke. Results of the multivariable adjusted models are indicated. Abbreviations: ECG, electrocardiogram; TIMI, thrombolysis in myocardial infarction; PCI, percutaneous coronary intervention; LVEF, left ventricular ejection fraction; LV, left ventricular; IABP, intra-aortic balloon pump; ACEi, angiotensin converting enzyme inhibitor; ARB, angiotensin II receptor blocker.
